# Supplementary material for: A nanobody suite for yeast scaffold nucleoporins provides details of the nuclear pore complex structure
Source: Nat Commun. 2020 Dec 2;11:6179. doi: 10.1038/s41467-020-19884-6 (PMC7710722; doi:10.1038/s41467-020-19884-6)
Supplement: Supplementary file 1 — Supplementary Information [file 41467_2020_19884_MOESM1_ESM.pdf]

Supplementary Information for:

A nanobody suite for yeast scaffold nucleoporins provides details of the Nuclear Pore Complex structure

Sarah A Nordeen[1], Kasper Andersen[1], Kevin E Knockenhauer[1], Jessica R Ingram[2], Hidde Ploegh[2], Thomas U Schwartz[1*]

[1]Department of Biology, Massachusetts Institute of Technology, Cambridge, Massachusetts, United States
[2]Boston Children's Hospital and Harvard Medical School, Boston, Massachusetts, United States
*corresponding author

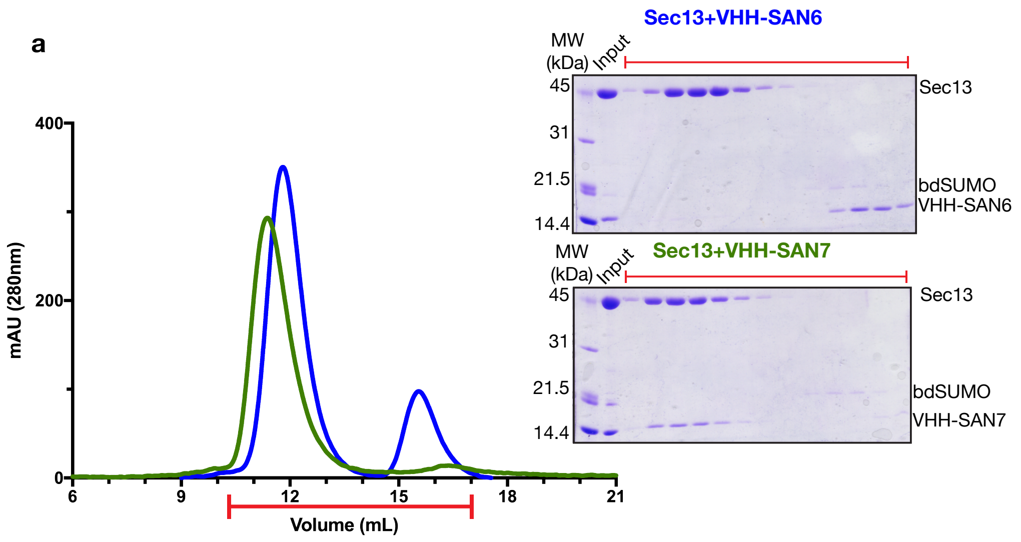

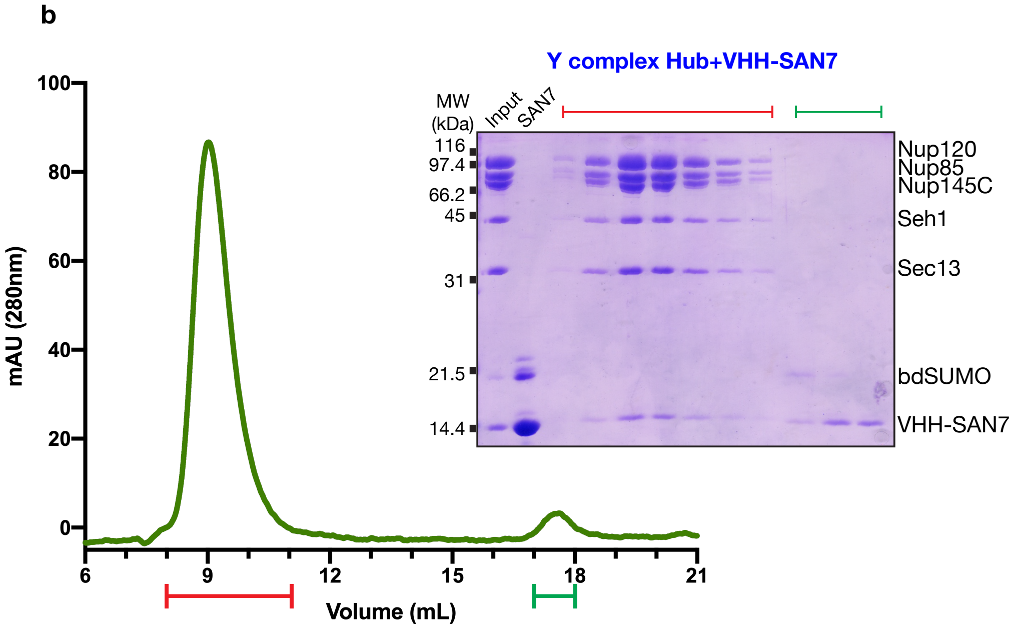

Supplementary Figure 1.

VHH-SAN7 binds Sec13 in the Y complex hub

(a) Size exclusion chromatography (SEC) of Sec13-Nup145C$_{blade}$ pre-incubated with VHH-SAN6/7 (1:1 molar ratio). SDS-PAGE analysis of SEC experiments for the fractions indicated. Co-migration of Sec13 and VHH-SAN7 indicates complex formation, whereas VHH-SAN6 migrates as a separate peak. (b) SEC with pre-incubated Y complex hub and VHH-SAN7 (1:2 molar ratio). SDS-PAGE analysis of the SEC experiment for the fractions indicated. Co-migration of the hub and VHH-SAN7 shows complex formation. Each panel is a representative experiment from two independent experiments.

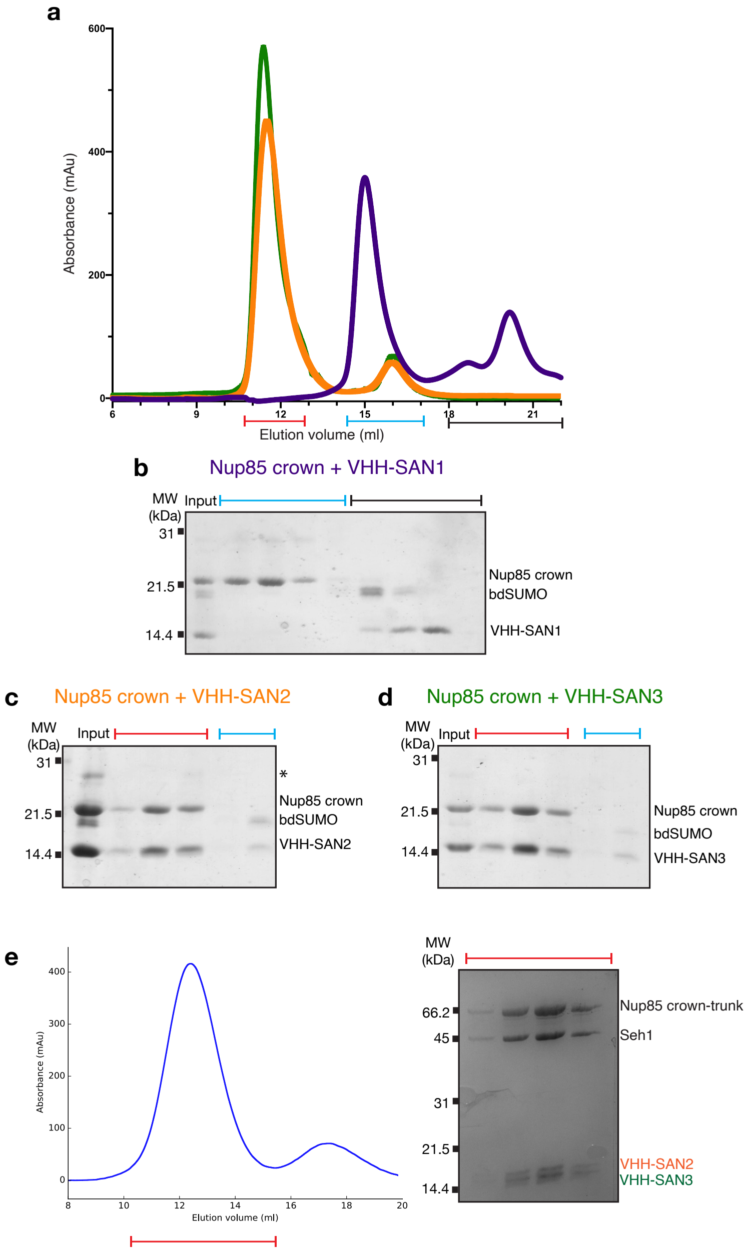

Supplementary Figure 2.

VHH-SAN2 and VHH-SAN3 bind the Nup85 crown module

(a) Size exclusion chromatography (SEC) of Nup85$_{crown}$ pre-incubated with VHH-SAN1 (purple) VHH-SAN2 (orange) VHH-SAN3 (green) (1:1.5 molar ratio), absorbance at 280 nm. (b, c, d) SDS-PAGE analysis of SEC experiments of the indicated fractions for (b) VHH-SAN1 (c) VHH-SAN2 (c) VHH-SAN3. * indicates an unknown contaminant. (e) SEC of Nup85$_{crown\text{-}trunk}$-Seh1 pre-incubated with VHH-SAN2/3 (1:1.5 molar ratio), absorbance at 280 nm. Left panel shows SDS-PAGE analysis of the indicated fractions. Each panel is a representative experiment from three independent experiments.

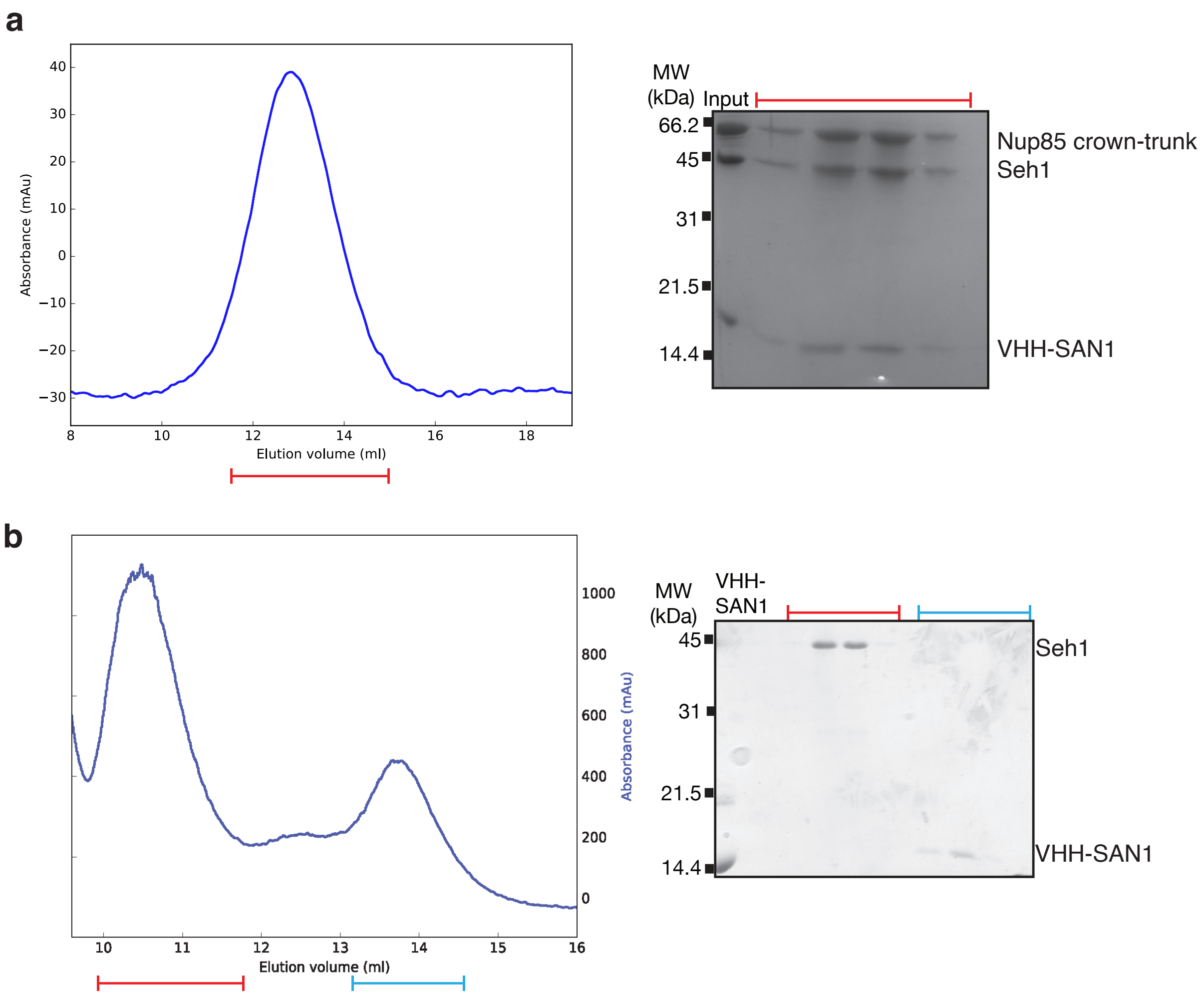

Supplementary Figure 3.

VHH-SAN1 binds the Nup85 trunk module

(a) Size exclusion chromatography (SEC) of $Nup85_{crown\text{-}trunk}$-Seh1 pre-incubated with VHH-SAN1 (1:1 molar ratio), absorbance at 280 nm. Left panel shows SDS-PAGE analysis of the indicated fractions. (b) SEC of Seh1 pre-incubated with VHH-SAN1 (1:1 molar ratio), absorbance at 220 nm. Left panel shows SDS-PAGE analysis of the indicated fractions.

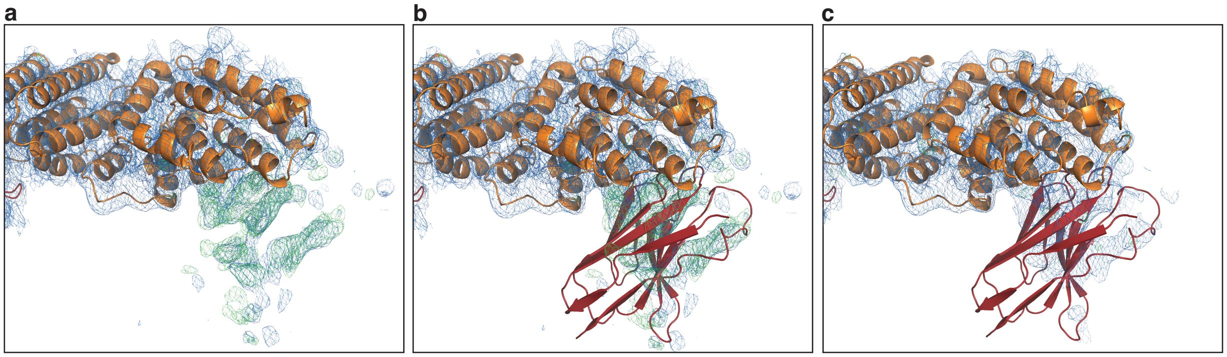

Supplementary Figure 4.

Difference density at the Nup85 crown

Electron density for the $Nup85_{crown}$ and VHH-SAN2. $2F_O$-F_C map contoured at 1σ is shown in blue, the F_O-F_C map contoured to 2.5σ shown in green. (a) Density after placing only Nup85-Seh1. (b) Rigid body placement of a VHH-SAN2 model. (c) Final density after refinement with VHH-SAN2. Residues outside of the density for VHH-SAN2 have an occupancy set to zero.

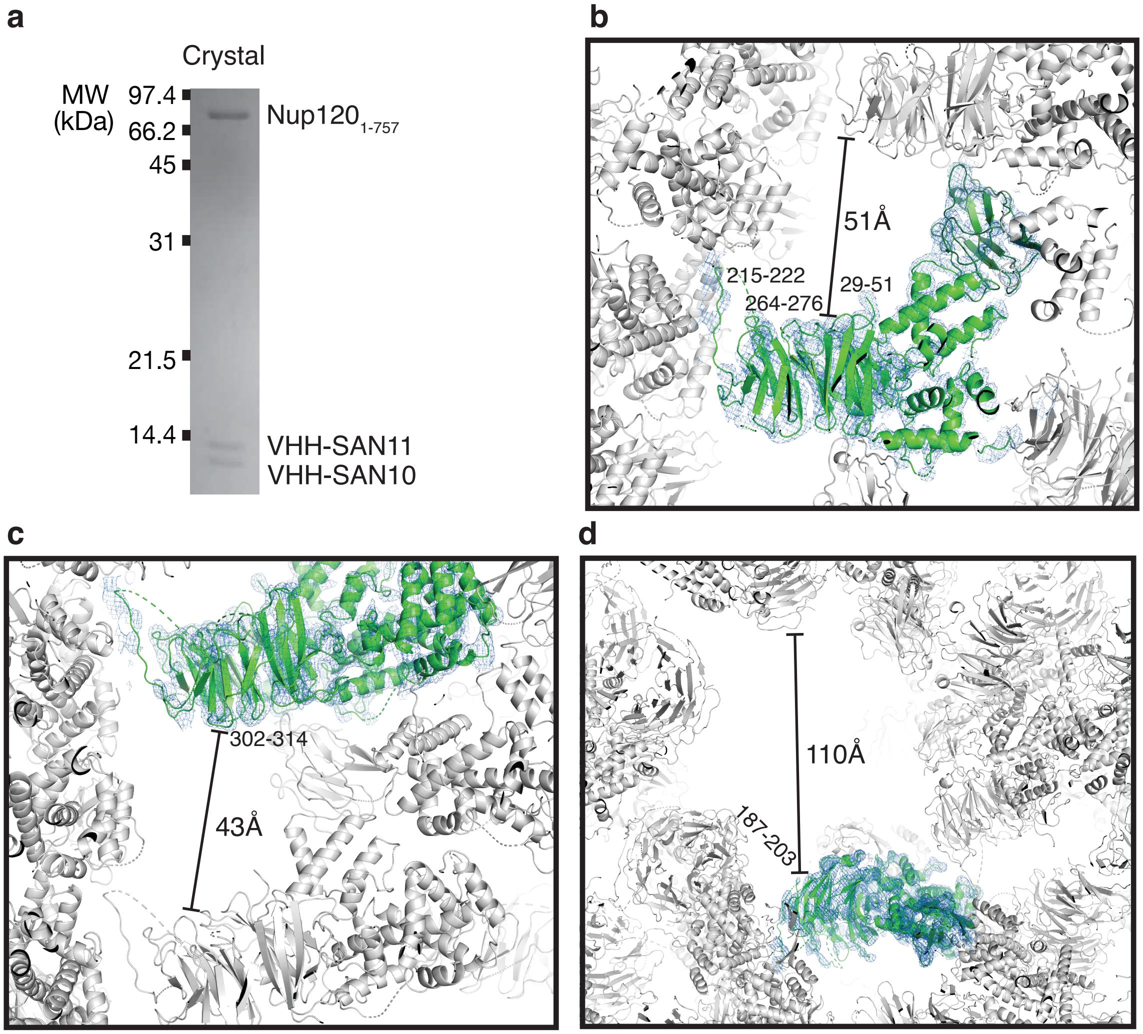

Supplementary Figure 5.

Crystal packing in Nup120-VHH-SAN10/11

(a) SDS-PAGE of Nup120$_{1\text{-}757}$-VHH-SAN10/11. Both nanobodies are present in the crystals. Image is representative of two independent experiments. (b, c, d) Views of crystal packing contacts and solvent channels. One copy of Nup120$_{1\text{-}757}$-VHH-SAN11 shown in green with 2F$_O$-F$_C$ density contoured to 1σ. Symmetry related molecules are shown in gray. Unbuilt loops lacking electron density are listed and distances between molecules are indicated.

Supplementary Table 1.
List of Primer Sequences for Cloning

Name	Forward primer (5'-3')	Reverse primer (5'-3')	Purpose
Nup133 FL insert	GTGGCTCAAGTGGATCCATGAGTGAAAAAAAAGTACATCTTCG	CCAGACTCGAGGGTACCTTAGTATTCTACAGTGTTGGTTTCATAGTTG	Amplify Nup133 for Gibson assembly
pAH His14bdSUMO nup vector open	CAACTATGAAACCAACACTGTAGAATACTAAGGTACCCTCGAGTCTGG	CGAAGATGTACTTTTTTTTCACTCATGGATCCACTTGAGCCAC	Amplify pAH His14bdSUMO vector for Gibson assembly
Nup133 NTD insert	GTTCTGTTCCAGGGGCCCGGATTCGATAATTCCAAGGTTTTCACAG	CCAGACTCGAGGGTACCTTACTACTCTTTCACAAAAAACTGTAGCACC	Amplify Nup133 NTD for Gibson assembly
pET28 vector open	GGTGCTACAGTTTTTTGTGAAAGAGTAGTAAGGTACCCTCGAGTCTGG	CTGTGAAAACCTTGGAATTATCGAATCCGGGCCCCTGGAACAGAAC	Amplify pET28 6His vector for Gibson assembly
Nanobody hit insert	GACTGGTGGCTCAAGTGGATCCATGCAGGTGCAGCTCGTG	ATGCGGCCGTGTACATTATCACTATTATGCGGCACGCGGTTCC	Amplify nanobody hit sequences for Gibson assembly
pAH His14bdSUMO nanobody vector open	GGAACCGCGTGCCGCATAATAGTGATAATGTACACGGCCGCAT	GCATGGATCCACTTGAGCCACCAGTC	Amplify pAH His14bdSUMO vector for Gibson assembly for nanobody expresion
Inverse add nano AviTag	CACAGAAAATCGAATGGCATGAATAATAGTGATAATGTACACGGCCGCAT	CTTCAAAGATGTCGTTCAGACCCGAGGAGACGGTGACCTGG	Add C-terminal AviTag to nanobody expression vector
Nup120 mut1	CTAGCGGTGGCTCTGAAGAATATTTGGCTAATTTAGAAACAATATTGAGAGAC	AGCCAGAGCTACCATTTTCACTCAATATTTGCTGTGCACG	Make $Nup120_{1\text{-}757}$ Δ431-439 (GGGSx2)
Nup120 mut2	GCTCAGGTGGTTCTGGCGGAAGTTACTTGAAAAGCCTAACGCG	CACCGCTGCCACCAGAACCGCCTAAACCCAACAAACCGCCATC	Make $Nup120_{1\text{-}757}$ Δ187-203 (GGSx5)
pGal, mKate2 vector open	CCAGGTCACCGTCTCCTCGTCGATATCAAGCTTAATGGTGAGCG	CACGAGCTGCACCTGCATTTTTAGCCTGCTTTTTTGTACAAACTTGTG	Amplify Gal promoter yeast expression vector
pGal nanobody insert	CACAAGTTTGTACAAAAAAGCAGGCTAAAAATGCAGGTGCAGCTCGTG	CGCTCACCATTAAGCTTGATATCGACGAGGAGACGGTGACCTGG	Amplify nanobody for Gibson assembly into yeast expression vector
Remove mKate2	TAACTCGAGTCATGTAATTAGTTATGTCACGC	CGAGGAGACGGTGACCTG	Remove mKate2 fusion from nanobody yeast expression
Nup120 promoter amp	GGAACAAAAGCTGGAGCTCTAGTAATGGTACCACTCCATTTGTTG	AATAGGTCAGTTGTGTACGAGTGAAATTGCG	Amplify 500bp upstream of Nup120 ORF in yeast
Yeast vector open	GATGGGCTGCAGGAATTC	TACTAGAGCTCCAGCTTTTGTTCC	Amplify yeast expression vector to swap Gal promoter to Nup120 promoter
p120 nanobody mKate2 insert	CACTCGTACACAACTGACCTATTATGCAGGTGCAGCTCG	GAATTCCTGCAGCCCATCTTATCTGTGCCCCAGTTTGCTAG	Amplify nanobody for Gibson assembly into Nup120 promoter yeast expression plasmid

p120 yeast vector open	AATAGGTCAGTTGTGTACGAGTGAAATTGCG	GATGGGCTGCAGGAATTC	Amplify Nup120 promoter yeast expression plasmid for nanobody Gibson assembly
